# Food Aroma Formation by Filamentous Fungi: Metabolic Pathways and Selected Applications, with Emphasis on Asian Fermented Foods

**DOI:** 10.3390/foods15213793

**Published:** 2026-10-23

**Authors:** Ling Xu, Jiali Li, Feng Wang, Jingya Qian, Shuhao Huo, Xianli Gao, Zhongyang Ding

**Affiliations:** 1 School of Food and Biological Engineering, Jiangsu University, Zhenjiang 212013, China; 2222518057@stmail.ujs.edu.cn (J.L.); fengwang@ujs.edu.cn (F.W.); qianjingya@ujs.edu.cn (J.Q.); huo@ujs.edu.cn (S.H.); gaoxianli@ujs.edu.cn (X.G.); 2 Key Laboratory of Carbohydrate Chemistry and Biotechnology, Ministry of Education, School of Biotechnology, Jiangnan University, Wuxi 214122, China; bioding@163.com

**Keywords:** fungi, aroma compounds, fermentation, flavor enhancement, volatile compound

## Abstract

Volatile compounds (VOCs) are important factors affecting the quality of fermented foods, acceptance of plant-based ingredients, and development value of natural flavors. Coupled with the increasing consumer demand for natural and clean-label foods, microbial fermentation has been promoted as a sustainable aroma production pathway. Compared with yeast, which mainly shapes flavor through ethanol fermentation, higher alcohols, and ester metabolism, basidiomycetes and filamentous fungi show more prominent extracellular enzyme secretion, complex substrate degradation, lipid oxidation, phenolic acid transformation, terpenoid transformation, and aromatic precursor biotransformation ability, which can synthesize diverse volatile organic compounds. This review focuses on the research progress in the production of volatile compounds by basidiomycetes and filamentous fungi (yeasts are excluded) for improvements in fermented flavor and reviews the groups, metabolic basis, fermentation process, and application in fermented food of aroma-producing fungi with selected application examples emphasizing Asian fermented foods. The challenges of fungal fermentation and the prospect of future research are also discussed here, providing a theoretical basis for the development of sustainable flavor ingredients.

## 1. Introduction

Food aroma is a human olfactory perception triggered by volatile compounds, which are composed of alcohols, esters, aldehydes, ketones, acids, lactones, terpenes, phenols, pyrazines, sulfur-containing compounds, and other volatile substances [1,2,3]. In this review, volatile compounds (VOCs) refer to all low-molecular-weight substances detectable by GC–MS, regardless of their sensory contribution. By contrast, the term aroma-active compounds is reserved exclusively for those volatiles whose sensory relevance has been confirmed by gas chromatography–olfactometry (GC–O), aroma recombination, or omission experiments. Not all volatile substances can form a perceptible aroma, and only aroma-active volatiles can produce significant sensory effects [4,5,6]. Among various sensory attributes, aroma plays a leading role in determining the overall flavor perception of food [7]. However, traditional natural aroma compounds mainly rely on plant extraction and chemical synthesis, and the plant sources are limited by season, region, raw material price, and extraction efficiency, while chemical synthesis products struggle to meet the requirements for natural sources and clean labels in some markets [8,9]. Therefore, the development of microbial fermentation using beans, fruit and vegetable residues, and edible fungus substrates as raw materials has received increasing attention, focusing on the advantages of resource utilization and specific biotransformation [10,11,12,13]. The microorganisms that produce aroma compounds through microbial fermentation biotechnology include bacteria (such as lactic and acetic acid bacteria) and fungi (such as yeasts, molds, and basidiomycetes), among which fungi have received extensive attention due to their unique metabolic ability [14,15,16].

Fungi (especially filamentous fungi and basidiomycetes) have different functional positioning in food fermentation systems compared to bacteria and yeasts and have unique advantages in producing aroma compounds [17,18]. *Aspergillus*, *Rhizopus*, basidiomycetes, and other filamentous fungi have stronger extracellular enzyme secretion ability and more complex secondary metabolic potential. They can decompose complex substrates into small molecular precursors that can be further transformed by amylase, protease, lipase, esterase, laccase, peroxidase, and glycosidase in solid, semi-solid, and liquid fermentation environments [19,20,21]. These precursors can enter the processes of sugar metabolism, organic acid formation, fatty acid oxidation, phenolic acid transformation, and terpenoid transformation [22], forming various types of volatile compounds with floral, fruity, cheese, chocolate-like, mushroom, and nutty sensory characteristics. Thus, fungi are important for flavor regulation in the biotransformation of soy sauce, douchi, tea, sufu, cheese, starter cultures, plant-based food, and agricultural by-products [23,24,25]. In addition, unlike bacteria and yeasts, filamentous fungi can produce aroma profiles that are highly similar to those of plants, which makes them particularly valuable in the production of natural flavors [26,27].

In recent years, significant progress has been made in the study of fungal aroma production, from traditional starters and naturally fermented foods to plant protein ingredients, plant-based cheese, edible fungal mycelium, tea fermentation, and fermented beverages [28,29], and several reviews have addressed specific aspects of fungal aroma production. For example, some have summarized the discovery of new aroma-producing fungal strains [30,31], and some focused on the metabolic pathways of aroma production by various fungi (such as *Aspergillus*, *Rhizopus*, and basidiomycetes) [32,33,34]. In addition, the optimization of the microbial fermentation process and the utilization of agricultural by-products have been reviewed [35,36,37]. However, the systematic integration of microbial resources, metabolic mechanisms, process optimization, and food applications has yet to be explored. Therefore, this paper reviews the fungal groups, metabolic basis, fermentation process, and application in fermented food of volatile compounds produced by filamentous fungi and basidiomycetes except yeast with the aim of providing a theoretical basis for the application of microbial fermentation technology in the food industry. At the same time, this field also faces certain challenges; for example, the molecular mechanism for controlling aroma formation has not been clearly explained, and there are great challenges to the amplification of solid-state fermentation processes. Finally, future development pathways for fungal fermentation technology are proposed at the end of this paper.

This work is a narrative review rather than a systematic review. Articles were retrieved from Web of Science, Scopus, and ScienceDirect, combining keyword searches: filamentous fungi, basidiomycetes, aroma compounds, volatile organic compounds, solid-state fermentation, submerged fermentation, and fermented food flavors. We screened peer-reviewed original research articles and existing reviews, prioritizing studies reporting fungal metabolic mechanisms, pure-culture experiments, and food-application evidence. The literature covers both recent advances and classical foundational studies. This review aims to bridge the knowledge gap by integrating fungal strain resources, metabolic mechanisms, fermentation process regulation, and real-world fermented food applications. Most previously published reviews only addressed isolated aspects, including strain screening, agro-industrial by-product valorization, or individual metabolic pathways.

## 2. Aroma-Producing Fungi and Their Flavor Functions

### 2.1. Aspergillus

*Aspergillus* is one of the most representative filamentous fungi in soy sauce, starter cultures, tea, and a variety of plant-based fermented foods, and its flavor function can be divided into three categories [33]. *Aspergillus oryzae* mainly undertakes saccharification, proteolysis, and plant protein modification, showing strong starch hydrolysis, protein modification, and flavor precursor release ability in quinoa, broad bean, and liquor systems [25]. *A. niger* participates in phenolic acid conversion and vanillin-related pathways. Studies on pumpkin peel fermentation have shown that it can connect food processing waste with bio-vanillin production [38]. *Eurotium cristatum* (syn. *Aspergillus cristatus*) is a key fungus in the fermentation of tea golden flowers, which can reshape the microbial community of tea and is related to changes in key volatile compounds such as benzaldehyde, linalool, methylsalicylic acid, β-ionone, and 1-octen-3-ol [39]. Studies on *Eurotium cristatum* have shown that the source of the strain affects the aroma classification of fermented tea. Studies on the fermentation of white tea have shown that *Eurotium cristatum* can shift the system from bacterial- to fungal-dominated and increase esters, ketones, and heterocyclic compounds [39]. Studies on dark tea further compared *Eurotium cristatum* strains from different regions and found that they could enhance floral, sweet, woody, and fruity aromas, but the key aroma components were different [40]. Strains such as *A. oryzae*, *A. niger*, and *Eurotium cristatum* usually release amino acids, reducing sugars, fatty acids, and glycosidically bound aroma precursors through extracellular hydrolases, thereby affecting the formation of subsequent alcohols, aldehydes, acids, and esters. In a system of soy sauce and starter cultures, the enzymatic hydrolysis of *Aspergillus* is closely related to the metabolism of amino acids, sugar alcohols, and fatty acids, which is an important part of the formation of soy sauce flavor, alcohol flavor, and flower flavor precursors [41,42].

### 2.2. Rhizopus

The core functions of *Rhizopus* are plant matrix saccharification, acid–ester balance, and lipid conversion [43]. In this review, *Rhizopus oligosporus* and *Rhizopus arrhizus* are carefully distinguished. These two strains are different in taxonomic status, zymography and niche. *Rhizopus oligosporus* is widely used in tianpei fermentation, known for its ability to produce esters, pyrazines and organic acids, especially in lipid metabolism. *R. arrhizus* (syn. *R. oryzae*) is commonly found in traditional rice wine koji (Jiuqu), which shows strong saccharification and acid protease activity, and contributes to the formation of esters, alcohols and organic acids in wine fermentation system. Researchers have compared *A. oryzae*- and *R. oligosporus*-fermented broad bean powder and found that *A. oryzae* is more conducive to protein increase and umami-related attribute formation, while *R. oligosporus* more significantly affects lipid metabolism and some aroma attributes [25]. In addition, free amino acids and umami were more prominent in *A. oryzae* samples, while esters and pyrazines were more abundant in *R. oligosporus* samples, indicating that *A. oryzae* and *R. oligosporus* have complementary values in the design of composite starter cultures [44]. The application of *R. oligosporus* in quinoa, faba bean, rice bran, and mycelium ingredients showed that *R. oligosporus* can simultaneously affect nutritional composition, anti-nutritional factors, functional properties, and volatiles [45]. Studies on the mycelium of *R. oligosporus* have shown that the biomass obtained via solid-state fermentation (SSF) and submerged fermentation (SMF) had different sensory attributes. SMF samples were rich in furfural derivatives, 2-phenylethanol, and fatty acids, featuring caramel, floral, and creamy sensory characteristics, respectively. The volatiles of SSF samples were lower, and their flavor was relatively neutral [43]. This difference indicates that the food application direction of the same fungal strain depends not only on the genetic background but also on the fermentation morphology and product positioning. The screening of *R. arrhizus* strains showed that there were significant differences in enzyme activity, antioxidant capacity, and volatile production among different strains, indicating that the *R. arrhizus* resources in traditional starter cultures could be developed into a directional flavor strain library [46].

### 2.3. Penicillium

The representative functions of *Penicillium* in this field focus on lipolysis and cheese flavor formation. The cheese aroma of *Penicillium* is the result of fatty acid precursors and lipases, and *Penicillium* does not automatically produce cheese flavor on any plant substrate [47]. In general, mold-ripened cheeses form unique and complex flavor characteristics during ripening. The main flavor compounds, including methyl ketones such as 2-heptanone and 2-nonanone, are aroma-active compounds produced through the lipolysis of mold, mainly from medium-chain saturated fatty acids. This enzymatic decomposition of milk fat contributes to the unique sensory characteristics of cheese during ripening [48]. Therefore, these key compounds can be used as indicators of cheese flavor development in mold-matured plant cheeses. *P. roqueforti* and *P. camemberti* have shown a clear precursor dependence in the study of fermented soymilk: if the soymilk system lacks short- and medium-chain fatty acid precursors, it is difficult to form key ketone flavor compounds such as 2-heptanone and 2-nonanone even if inoculated with *Penicillium*; following the addition of coconut oil, mold lipase can convert fatty acid precursors into target flavors [48]. These results have important implications for the development of plant-based cheese: flavor failure is often not simply due to incorrect strain selection but also the lack of a precursor design.

### 2.4. Basidiomycetes

Basidiomycetes are one of the most expansive groups of aroma-producing non-yeast fungi. *Mycetinis scorodonius* can produce chocolate- and malt-like aromas in green tea infusion, and its process robustness has been verified via gas chromatography–mass spectrometer–olfactometer (DI-SBSE-GC-MS-O) and laboratory bioreactor transfer [49]. Monokaryotic strains of *Pleurotus sapidus* can produce p-anisaldehyde and related aromatic compounds in the sidestream of citrus, forming a fennel and woody aroma [50]. The edible and medicinal fungus *Wolfiporia cocos* can be used with black currant pomace to develop fermented beverages and form unique flavors. Linalool (citrus), geraniol (flowery), phenylacetic acid (honey), methyl phenylacetate (honey), eugenol (clove), and 2-phenylethanol (rose) are produced during the fermentation process [51]. The basidiomycete *Auriporia aurulenta* can be used with fruit residues to form aromatic volatiles dominated by esters and alcohols, and its potential comes from its oxidoreductase system and complex aromatic precursor conversion ability [52]. Agricultural–industrial waste residues can be used as a source of biological spices produced by basidiomycetes. Among them, *Pleurotus*, *Bjerkandera*, *Marasmius*, *Trametes*, and *Pycnoporus* have the potential to convert low-value residues into benzaldehyde, benzyl alcohol, 2-phenylethanol, terpenoids, and C8 compounds [18,53]. In particular, white-rot basidiomycetes can produce aroma compounds with commercial value, such as vanillin and benzaldehyde. White-rot basidiomycetes can convert vanillic acid into vanillin, which has a pleasant aroma. Benzaldehyde is the second largest aromatic compound with the aroma of apricot, cherry, and almond. Several basidiomycetes, such as *Poria subacida*, *Dichomitus squalens*, *Ischnoderma resinosum*, *Sarcodontia setosa*, and *Hericium erinaceus*, have been found to produce benzaldehyde [54,55]. Different from *Aspergillus* and *Rhizopus* (filamentous fungi responsible for saccharification, proteolysis, and acidification), basidiomycetes are more suitable for the production of natural flavors and fermented beverages [56].

### 2.5. Other Filamentous Fungi

Red filamentous fungi have both flavor and color value in traditional soybean products and grain fermentation. HongJun Tofu’s research showed that *Neurospora crassa* can provide a traditional mushroom flavor substrate, and compound fermentation can further increase esters, alcohols, acids, ketones, and 5′-nucleotides, thereby enhancing umami and reducing bitterness [35]. It should be noted that this study focuses on a multi-bacterial complex system, and *Meyerozyma* and lactic acid bacteria are also involved in flavor formation, so all flavor improvement cannot be attributed to *N. crassa*. In a study on Daqu and Jiang-flavor baijiu, *Monascus*, *Thermoaspergillus*, *Thermomyces*, and *Aspergillus* are related to the formation of alcohols, aldehydes, acids, esters, pyrazines, furans, phenols, and sulfur compounds [57]. Complex fungal networks are particularly important in traditional wine fermentation. Artificial Daqu has higher fungal diversity and richer ester/alcohol volatiles than mechanical Daqu, but mechanical Daqu has advantages in liquefaction capacity and standardization [58]. In addition, the use of fortified Daqu can improve the quality of Luzhou-flavor baijiu, increase the content of ethyl caproate, and adjust the proportion of other flavor components [59]. Among them, it should be noted that the yeasts reported in Daqu, *Millerozyma*, and other yeasts belong to the yeast genus, which are not within the core scope of this review. They are only mentioned as background members of complex priming ecosystems. These results indicate that the traditional fermentation system needs to find a balance between complex ecology and process controllability. Table 1 lists the main fungal groups that produce aroma compounds.

## 3. Metabolic Basis of Fungal Aroma Production

In this review, evidence for fungal-related aroma change is categorized into four levels: (i) direct aroma-compound biosynthesis proven via pure-culture or controlled biotransformation assays [49,50,52]; (ii) fungal-mediated release or bioconversion of aroma precursors [62]; (iii) indirect aroma modulation within multi-species mixed-microbial communities [63,64]; and (iv) associative evidence derived only from statistical correlation between fungal abundance and volatile profiles [65]. Correlation alone cannot serve as definitive proof for direct fungal biosynthesis of target aroma substances.

**Figure 1:**
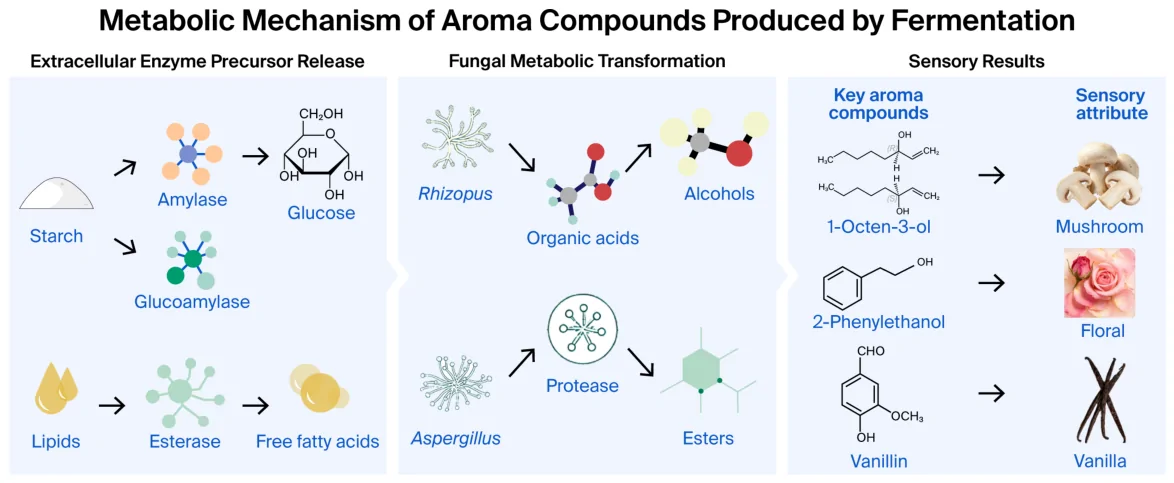
The metabolic mechanism of volatile compounds produced via fungal fermentation.

The basis of aroma production via fungal fermentation lies in its extracellular enzyme secretion ability and secondary metabolic potential. The mycelia of *Aspergillus*, *Rhizopus*, *Monascus*, *Eurotium cristatum*, and edible fungi can secrete protease, glucoamylase, cellulase, lipase, glycoside hydrolase, and tannase in solid, semi-solid, or liquid systems to promote the degradation and release of protein, starch, polyphenols, lipids, and glycoside-bound precursors [66,67]. These precursors are further involved in amino acid conversion, fatty acid oxidation, esterification, terpenoid metabolism, and Maillard reactions to form alcohols, esters, aldehydes, ketones, terpenoids, pyrazines, and C8 mushroom-like volatile compounds [27,68]. Fungal aroma production has dual attributes: on the one hand, it increases the complexity of food aroma through enzymatic degradation and metabolic transformation [69]; on the other hand, it alters non-volatile components such as proteins, fibers, polyphenols, anti-nutritional factors, and functional metabolites [70]. The metabolic mechanism of volatile compounds produced via fermentation is shown in Figure 1.

### 3.1. Extracellular Enzymes Release Flavor Precursors

The production of aroma by fungi first depends on the decomposition of complex food substrates by extracellular enzymes. Amylase and glucoamylase release glucose, maltose, and other fermentable sugars [1]; protease releases free amino acids and small peptides [33]; lipase releases free fatty acids [46]; esterases and glycosidases are involved in the hydrolysis of bound aroma precursors [48]; and laccase and peroxidase are involved in the redox of aromatic compounds [60]. These reactions determine the precursor pool of subsequent aroma formation, which is the key link connecting the matrix composition and volatile product spectrum [36]. Among them, the endopeptidase, carboxypeptidase, and aminopeptidase (exopeptidase) work together to hydrolyze the storage protein into free L-glutamic acid and 5′ -nucleotide. These two key compounds give soybean, broad bean, and quinoa a unique umami flavor [71]. *Rhizopus* can produce amylase and glucoamylase, decompose starch into glucose, and produce flavor-enhancing substances such as lactic acid and higher alcohols, creating the unique aroma and taste of traditional Chinese alcoholic beverages [72,73]. Moreover, different strains of *Rhizopus* have obvious differences in the activities of acid protease, amylase, glucoamylase, and esterase, corresponding to the formation ability of different volatiles and key aroma-active compounds [74]. *A. oryzae* and *R. oligosporus* can secrete a large number of enzymes such as proteases, amylases, cellulases, and esterases, which can decompose proteins, polysaccharides, and lipids [75]. When fermenting quinoa and broad beans, the nutritional and flavor properties of plant substrates can be improved by changing the composition of proteins, starches, oligosaccharides, organic acids, and volatiles [33]. Relevant studies have indicated that the aroma formation of fungi cannot be explained only with regard to the end-point volatiles; the release process of enzymatic precursors should also be considered. The laccase system of white-rot fungi further expanded the relationship between fungal enzyme systems and aroma production. *Trametes versicolor* and *Pycnoporus sanguineus* can be induced by farnesol to produce high laccase activity in solid-state fermentation of pineapple waste, which provides an enzymatic basis for the conversion of aromatic precursors [60,76].

### 3.2. Carbohydrate and Organic Acid Pathway

*Aspergillus* and *Rhizopus* are often the core microbiota involved in saccharification, acidification, and precursor release in the fermentation of cereals, legumes, and plant proteins. *A. oryzae* has shown strong saccharification and protein modification ability in a system of sweet wine, quinoa, and broad bean, improving the available sugar and amino nitrogen and promoting the formation of aldehydes, acids, and esters [33,77]. A comparative study between *A. oryzae* and *Bacillus amyloliquefaciens* showed that the *A. oryzae* group had higher saccharification efficiency and formed more aldehydes related to cocoa, malt, and floral aroma. *R. oligosporus* have demonstrated potential to produce organic acids, alcohols, esters, and many heterocyclic compounds in rice wine, quinoa, broad bean, rice bran, and mycelium ingredients [31,43]. Colored rice bran contains bioactive substances such as phenolic acids, flavonoids, anthocyanins, proanthocyanidins, tocopherols, tocotrienols, and phytic acid [78]. After fermentation with *R. oligosporus*, the types of volatiles increased significantly. Esters, aldehydes, alcohols, ketones, acids, phenols, furans, lactones, terpenes, and pyrazines together formed fat, fermentation, acid, nuts, and baked aromas [45,79]. These results suggest that cereal and legume by-products serve not only as nutritional matrices but also as a compound precursor pool for saccharification, amino acid release, and volatile production.

### 3.3. Lipid Oxidation and Methyl Ketone Pathway

Lipids and their hydrolysates are important precursors of cheese, mushroom, and fat flavors, as well as some fruit flavors. *P. roqueforti* and *P. camemberti* struggle to form typical cheese flavor due to insufficient short- and medium-chain fatty acids (C6-C10, e.g., caproic, caprylic, and capric acids) in an ordinary soymilk matrix. When the system is supplemented with fat sources rich in chain fatty acids such as coconut oil, *P. roqueforti* can generate key methyl ketones such as 2-heptanone and 2-nonanone through lipase and subsequent oxidation reactions and reduce the beany flavor [48,80]. This result indicates that the aroma function of fungi must be matched with the substrate precursor, and the inoculation of functional fungi (or starter cultures) alone cannot guarantee the formation of target aroma. When mixed lactic acid bacteria were used to ferment edible juice (*Hericium erinaceus* and *Tremella fuciformis*), the content of C8 compounds was significantly reduced, and the unwelcome mushroom flavor was reduced, thereby improving the overall aroma. C8 compounds include 1-octen-3-ol, 1-octen-3-one, 3-octanol, and 3-octanone, derived from the enzymatic oxidation of linoleic acid through the lipoxygenase/hydroperoxide lyase pathway, but it should be noted that the exact metabolic pathway details may vary from fungal species. Linoleic acid was first oxidized with lipoxygenase to 10-hydroperoxide, and then cleaved with hydroperoxide lyase to produce 1-octen-3-ol and 10-oxo-trans-8-decenoic acid. *P. ostreatus* can produce more C8-related compounds in Amaranth Seed-Based Media, such as 1-octen-3-ol, 1-octen-3-one, 3-octanone, and 3-octanol, producing mushroom, green, and fat-like sensory notes [81]. In Bambara Groundnut-Based Media, C8 compounds are relatively weakened, but malt, nuts, fermentation, and slight floral characteristics are enhanced [81]. Therefore, lipid composition not only determines the formation of monomeric volatiles but also affects the overall sensory type of mycelium ingredients.

### 3.4. Amino Acid Decomposition and Aromatic Compounds

Branched-chain and aromatic amino acids can form aldehydes, alcohols, acids, and esters through transamination, decarboxylation, reduction, and oxidation reactions [56]. Such reactions are often related to malt, nut, honey, flower, and fermented sensory notes. *P. ostreatus* produced more aldehydes and higher alcohols related to Ehrlich-type amino acid decomposition in the Bambara Groundnut-Based Media, indicating that the amino acid composition of the medium can regulate the aroma direction of the mycelium [82]. The metabolism of L-phenylalanine and L-tyrosine in aromatic amino acids produces a variety of important aroma-active compounds, including p-anisaldehyde, 2-phenylethanol, benzaldehyde, and vanillin. Among them, 2-phenylethanol is a rose-flavored aromatic alcohol produced from L-phenylalanine through the Ehrlich pathway, and the biotransformation from ferulic acid to vanillin is not uniformly linear in all fungal groups [83,84]; it is first converted from ferulic acid to vanillic acid, and then reduced to vanillin. Basidiomycete *Auriporia aurulenta* can produce floral and sweet volatile compounds represented by 2-phenylethanol and 2-phenylethyl acetate when using fruit residues for liquid fermentation [52,85]. These products are usually closely related to aromatic amino acid precursors, indicating that amino acids, sugars, and phenolic components in agricultural by-products can become high-value aroma production entrances [86]. In addition, solid-state fermentation of vegetable waste also lists 2-phenylethanol as one of the target aroma-active compounds with commercial potential in by-product fermentation [37].

## 4. Fermentation Process and Aroma Regulation

### 4.1. Solid-State Fermentation

Solid-state fermentation (SSF) is suitable for filamentous fungi to grow and secrete extracellular enzymes in low-free-water environments, especially for solid or semi-solid substrates such as cereals, beans, plant powder, rice bran, bean dregs, by-products of fruits and vegetables, and koji, so it has become the preferred method for fungal aroma production [37]. SSF features high substrate concentration and low water activity, generating water-related and mechanical stresses that mimic the natural ecological niche of filamentous fungi [87,88]. Such microenvironmental conditions favor the secretion of diverse extracellular enzymes and the biosynthesis of complex secondary metabolites [89,90]. The advantages of solid-state fermentation include its high substrate concentration, use of less water, easy simulation of the traditional food niche, and ability to induce extracellular enzyme release and local microenvironment formation [91]. At the same time, microorganisms grow on wet solid substrates without free water, which can promote the production of complex secondary metabolites (including volatile aroma compounds), and they have been successfully applied to the aroma production of various substrates [91,92]. For example, four filamentous fungi were used for the SSF of nine different agro-industrial by-products, and hundreds of volatile compounds were produced, including 1-octen-3-ol, phenylacetaldehyde, and phenylethanol. The yield of commercially related aroma-active compounds reached 1.493 g/kg, 1.297 g/kg, and 0.970 g/kg, respectively [36]. In addition, the SSF of broad bean and quinoa flours with *A. oryzae* and *R. oligosporus* can simultaneously change the nutritional composition, anti-nutritional factors, functional properties, and volatile profile [33]. However, due to the uneven distribution of mass transfer, heat transfer, and water in the solid matrix, fermentation amplification often faces problems such as batch differences, local overheating, and difficulty with online monitoring, limiting the application of SSF [75]. SSF intrinsically consumes less bulk water than submerged fermentation. However, downstream separation and purification of target volatile compounds from solid matrix may increase overall process-water demand. Although the screening of by-product SSF can quickly find a large number of potential aroma combinations, it is still necessary to solve the problems of parameter optimization, yield stability, and may require more complex downstream separation from screening to industrialization [14,93].

### 4.2. Submerged Fermentation

Submerged fermentation (SMF) is more suitable for controllable mycelium production, fermented beverages, and natural flavor processes. Mycelium produced via liquid fermentation is rich in a variety of bioactive compounds and may also have unique functional properties similar to fresh edible fungi, such as high protein and carbohydrate content, low fat and dietary fiber content, and medicinal use [94,95]. Unlike static culture, shake flask stirring can enhance the transfer, mixing, and shearing of oxygen, stimulate interaction with substrate components, and regulate the metabolism of fungi in a manner that static culture cannot capture [34]. The SMF of basidiomycetes would be closer to the biological manufacturing of natural aroma-active compounds. Among them, *Mycetinis scorodonius* is used with fermented green tea to produce a chocolate-like aroma [49]; *Auriporia aurulenta* is used with fruit residues to produce a floral fragrance, and *Wolfiporia cocos* ferments black currant pomace to form a beverage flavor [51]; edible fungi such as *Morchella*, *Tuber*, *Lentinula*, and *Agaricus* can produce mycelia and species-specific aromas in carbon source waste streams [94]. Compared with SSF, SMF has the advantages of parameter control, stirring, mass transfer, scale-up, and product recovery and is more suitable for the production of natural flavors and mycelium ingredients. By comparing the biomass produced by *R. oligosporus* in SSF and SMF, phenylethanol, ergosterol, and other components related to floral, umami, and meaty taste were found to be more prominent in SMF samples [43]. These results suggest that mycelium fermentation has potential in the development of plant-based foods, fungal proteins, and functional ingredients, but its application effect depends on the strain, fermentation method, and target food scenario. Therefore, the fermentation form should be selected according to the final product target. SSF can be given priority for the improvement of flavor-enhancing seasoning and traditional food, while SMF can be given priority for the production of high-protein mycelia, fermented beverages, and monomer aroma.

### 4.3. Mixed Fermentation

Traditional fermented food is usually not driven by a single fungus, but a complex ecological process of bacteria, fungi, environmental microorganisms, and physical and chemical factors [96,97]. In soy sauce koji-making, sufu, liquor, vinegar, and tea systems, microorganisms affect flavor through carbohydrate degradation, nitrogen-containing compound conversion, organic acid formation, and volatile substance synthesis [98,99,100]. Studies on soy sauce koji-making have shown that bacteria such as *Weissella* are related to flavor substances such as amino acids and ethyl acetate, indicating that bacteria are also involved in the flavor shaping of Aspergillus-dominated systems [101]. In addition, the co-fermentation of *Lactiplantibacillus plantarum* (formerly *Lactobacillus plantarum*), *M. guilliermondii*, and *N. crassa* significantly enriched the volatile flavor profile of HongJun Tofu compared with the single-strain fermentation using *N. crassa* alone [35]. In liquor production, fortified Daqu containing *B. subtilis*, *S. epidermidis*, and *M. farinosa* significantly improved the ester profile of Luzhou Laojiao Flavor Liquor [59]. The results showed that *Lactiplantibacillus* was positively correlated with ethyl acetate, while *Bacillus* and *Aspergillus* were positively correlated with beneficial flavor compounds. According to the study of summer black tea fermentation, the microbial community and volatile metabolites changed significantly, which increased the sweetness, alcohol, and smoothness of summer black tea, reduced the bitterness, transformed the aroma, and finally significantly improved the overall quality [102]. Although these studies are not all centered on fungi, they provide a reference for the functional boundary of non-yeast fungi in real fermentation ecology. Table 2 lists the typical volatile compounds, precursor sources, and fermentation forms of each fungus.

## 5. Application of Fungi in Fermented Food-Flavor-Forming Species

### 5.1. Liquor Fermentation System

As one of the six major distilled liquors in the world, liquor relies on complex microbial communities to form flavor. Different fungal communities drive the characteristic aroma profiles of different types of liquor [103]. Among them, starter cultures, Daqu, and fermented grains are important sources of non-yeast fungal food fermentation functions [104]. Screening of *R. arrhizus* starter strains showed that there were *Rhizopus* resources in traditional rice wine starters that could be used for saccharification, esterification, and flavor regulation [46]. *Rhizopus* resources significantly enhanced the aroma and flavor of yellow rice wine by producing basic volatile compounds (esters and alcohols) and key bioactive compounds (phenols and flavonoids) [105]. In a comparative study on artificial and mechanical Daqu, artificial Daqu has richer fungal community diversity and wider volatile compounds, especially esters and alcohols, which are crucial to the flavor characteristics of liquor [58,106]. The results showed that the reducing sugar content of artificial Daqu was 0.24% higher than that of mechanical Daqu, the water retention was 8.3% higher, and the acidity was increased by 0.10 units [58]. In contrast, the mechanically produced Daqu showed stronger liquefaction ability, but the fermentation ability was reduced. A study on fortified Daqu mentioned that exogenous functional bacteria and the ecological regulation of quyao could improve the ester spectrum and enhance the fruity and sweet aroma of Luzhou Laojiao Flavor Liquor. The results showed that fortified Daqu reduced the abundance of *Lactiplantibacillus*, increased the populations of *Bacillus* and *Aspergillus*, and significantly improved the ester spectrum. Ethyl caproate increased by 27.2%, while ethyl acetate decreased by 26.6%, resulting in a change in the fungal network [59,107]. The study of Maotai-flavor liquor further showed that *Aspergillus* and unclassified *Dipodascaceae* constituted the core fungal genus, significantly enhancing the production of key flavor compounds, including alcohols, acids, and esters, compared with the pure culture (*Zygosaccharomyces* and *Millerozyma* are background members outside the core review scope) [57]. A study on high-temperature Daqu connected the internal heterogeneity of Daqu, the aroma of base liquor, and the metabolic pathway of fatty acids, suggesting that different layers and types of Daqu may affect the style of base liquor through the transmission of key compounds [61,108]. In general, the contribution of non-yeast fungi in the traditional wine system features a complex community, strong interaction, and difficult causal verification (associative correlation-level evidence, requiring pure-culture validation).

### 5.2. Tea Fermentation System

Tea fermentation provides a relatively clear model system for the flavor function of *Eurotium cristatum*. Studies on white tea have shown that *Eurotium cristatum* fermentation can shift the microbial community from bacterial dominance to fungal dominance, reshaping the microbial community and increasing the fungal abundance from 0.43 to 64.94% [39,109]. At the same time, esters, ketones, and heterocyclic compounds were added; key volatile compounds such as benzaldehyde, ethyl 2-methylbutyrate, 1-octen-3-ol, and some ethyl esters were observed to contribute significantly to the aroma [109]. Metagenomic functional annotations have also revealed that Aspergillus cristatus fermentation can enhance the abundance of genes related to energy production and conversion, while reducing the abundance of antibiotic resistance genes and virulence factor genes [39]. In addition, *Eurotium cristatum* strains from different regions (Shaanxi, Sichuan, and Hunan) were compared, revealing that the strains from the three regions could enhance the floral, sweet, woody, and fruity aroma, but the combination of key aroma-active compounds was different, which mainly affected the aroma characteristics of dark tea after fermentation through changes in the composition and concentration of key aroma-active compounds [40]. Among them, several aroma-active compounds such as linalool, nonanal, 1-octen-3-ol, 6-methyl-5-hepten-2-one, β-ionone, and coumarin are contained in all samples, but their relative contents are different in fermented dark tea, resulting in different sensory results [110,111]. In addition, *A. niger* also showed a clear potential for flavor improvement in the fermentation of tea and tea by-products. Studies have found that *A. niger* and *Trichoderma koningii* SSF of tea residue can increase the content of amino acids, aromatic amino acids, and functional components, providing evidence for the resource utilization of tea by-products [21]. In addition, a decrease in ester catechins and an increase in linalool and its oxides were observed in summer green tea fermentation, indicating that *A. niger* fermentation could improve the quality of low-value tea resources by reducing bitterness-related components and enhancing floral volatile compounds [112]. The novelty of tea fermentation is that the source of strains can be an important variable for flavor typing. From the perspective of aroma design, different *Eurotium cristatum* strains may correspond to different terpenoids, aldehydes, esters, and ketones. Therefore, tea fungal fermentation can be shifted from empirical inoculation to strain screening and targeted aroma directional fermentation in the future.

### 5.3. Fermentation of Soybean Products

Douchi and HongJun Tofu represent the transition scenario between traditional food and new ingredients [113]. In traditional *Aspergillus* tofu production, the development and succession of microbial communities have a significant impact on the formation of and changes in characteristic volatile compounds [114]. *Aspergillus* was dominant in the early stage of fermentation, and the main flavor substances were soybean-specific alcohols and aldehydes, including 1-hexanol, 1-octen-3-ol, and nonanal. Later in the process, bacteria and yeast were enhanced, producing important flavor substances such as esters, ketones, and phenols, including guaiacol (2-methoxyphenol) and 3-octanone [115]. Mold is dominant at the pre-fermentation stage, and the number of bacteria increases rapidly at the post-fermentation stage. Correlation analysis between fungi and flavor substances reveals a higher correlation than with bacteria, which is an important reason for the formation of tofu flavor [113,114]. HongJun Tofu is a traditional fermentation substrate based on *N. crassa*. After multi-strain fermentation, the volatile profile is characterized by fruit, mushroom, flower, and nut notes. HongJun Tofu has less bitterness, increased freshness, rich taste, and no acidity [35,116]. However, it should be noted that reference 116 concerns a co-fermented milk beverage rather than Hong Jun Tofu; the discussion of the sensory properties of HongJun Tofu is therefore based on reference 35. In addition, solid-phase microextraction–gas chromatography–mass spectrometry (SPME-GC-MS) results showed that Hong Jun Tofu was rich in floral and fruit volatiles, including mushroom-characteristic 1-octen-3-ol and floral-scented 2-phenylethanol [35]. For the study of the natural fermentation of sufu, the results showed that bacteria and fungi were involved in the degradation of carbon and nitrogen sources and the formation of volatile aroma. A total of 19 compounds were identified as characteristic flavor compounds (taste-active and aroma-active precursors), including 6 amino acids, 3 organic acids, and 10 volatile aroma-active compounds [98,117]. These food systems usually contain a complex interaction among fungi, bacteria, and yeast. Dou chi and Hong Jun Tofu can provide evidence for traditional fermentation ecology and sensory improvement, but to prove that a non-yeast fungus directly produces a specific aroma-active compounds, pure-culture, synthetic community, or metabolic tracking experiments are still required.

### 5.4. Other Fermented Foods

**Figure 2:**
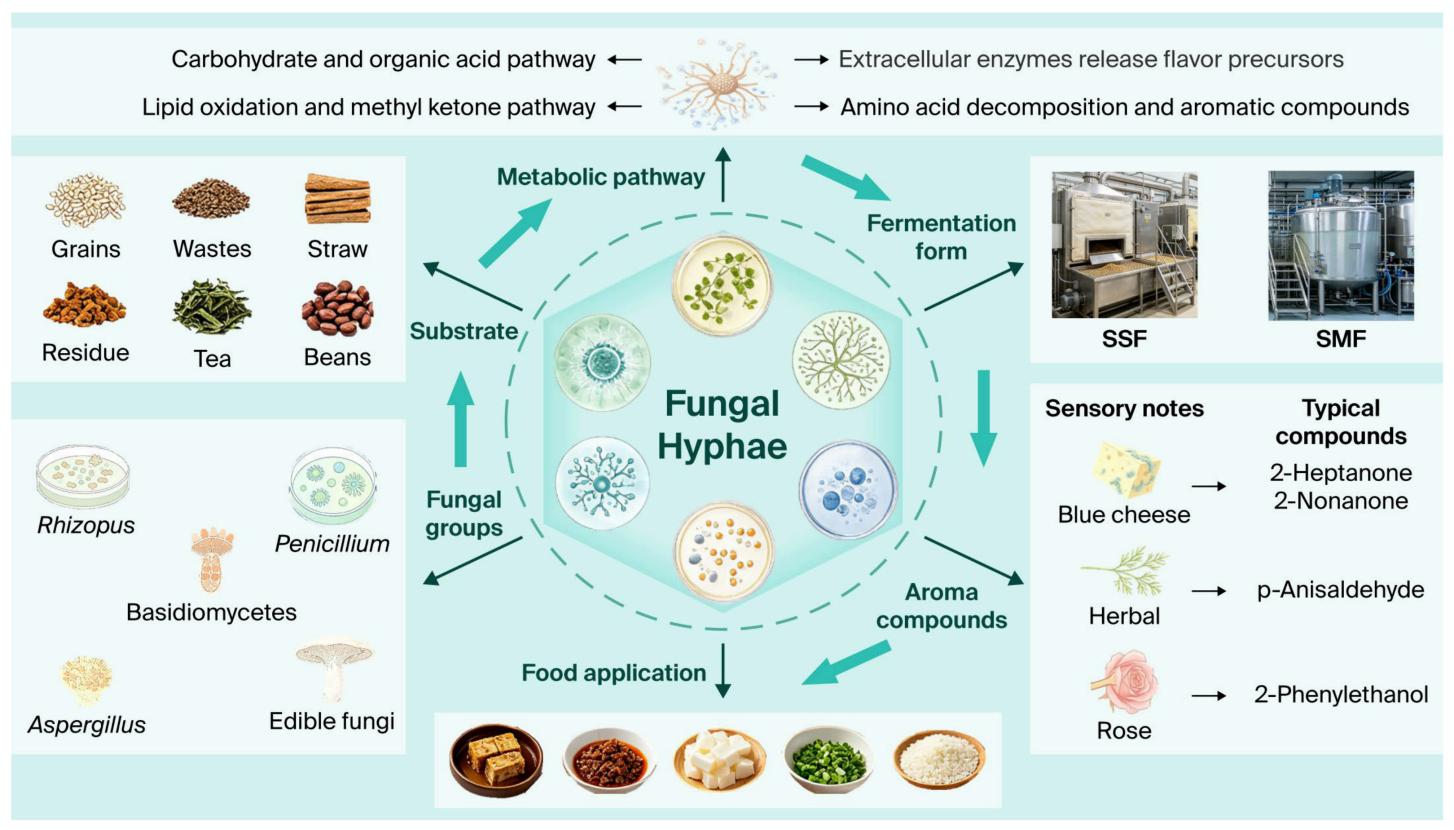
Diagram of logical relationships of fungal groups, substrates, fermentation forms, metabolic pathways, volatile compounds, and food applications.

In addition to the mentioned food systems, other fermented foods include plant protein ingredients, edible fungus juice, and mushroom food [118]. Among them, plant protein ingredients often face problems, including beany, bitter, grassy, insufficient functional properties, and nutritional limitations [119]. However, *A. oryzae* and *R. oligosporus* can change the composition of protein, fat, starch, oligosaccharides, organic acids, and volatiles and produce volatile compounds such as acids, aldehydes, ketones, esters, and lactones [25]. At the same time, when *R. oligosporus* mycelium was used as an alternative protein, SSF and SMF yielded differences in flavor intensity and formula applicability [43]. In addition, the volatiles of colored rice bran increased significantly after fermentation with *R. oligosporus*, which also showed that cereal by-products could be converted into more flavorful ingredients via fungal fermentation [45]. Studies on *P. ostreatus* mycelium have shown that the amino acid and lipid composition of the culture medium can direct the mycelium aroma to the C8 mushroom or Ehrlich malt/nutty aroma [81]. These studies suggest that the flavor improvement of plant-based ingredients should not only rely on post-addition flavors but also on endogenous flavor reconstruction through fungal fermentation and precursor design [120]. A study on the fermentation of mixed lactic acid bacteria in edible fungi juice showed that the compound juice was prepared via mixed-strain fermentation, with *Hericium erinaceus* and *Tremella fuciformis* as raw materials. The mixed fermentation of lactic acid bacteria could enrich the aroma characteristics of heat-clearing and dealcoholization, reduce the unpleasant odors such as nonanal and 1-octen-3-ol, and increase floral aroma components such as ethyl acetate [121]. Aroma precursors in edible fungi substrate can change dynamically during lactic acid bacteria fermentation, but the main fermentation body is lactic acid bacteria, so it is more suitable as the boundary evidence of “flavor transformation of edible fungi substrate” [122]. Studies on fermented mushrooms have shown that mushroom fermentation can improve preservation, sensory, and nutritional characteristics, but industrial-scale applications are still limited by pretreatment, cost, safety, and consumer preferences [123]. The logical relationship between fungal groups, substrates, fermentation morphology, metabolic pathways, volatile compounds, and food applications is shown in Figure 2.

## 6. Conclusions

Fungal fermentation is a promising and sustainable method for the production of natural aroma-active compounds. In particular, filamentous fungi and basidiomycetes have diverse metabolic capabilities and can synthesize a variety of volatile organic compounds, including C8 compounds, terpenes, aromatic aldehydes and alcohols, lactones, and pyrazines. Significant progress has been made in understanding the diversity of aroma-producing fungi, the metabolic pathways of aroma formation, and fermentation process optimization. Existing studies have shown that *Aspergillus*, *Rhizopus*, *Penicillium*, basidiomycetes, and other filamentous fungi can explain the release of precursors through extracellular enzymes and form diverse volatile compounds through sugar metabolism, organic acid production, lipid oxidation, and other pathways. A circular economy method has also been proposed for the resource utilization of agro-industrial by-products as fermentation substrates, which also solves the problems of waste management and sustainable aroma production: *Eurotium cristatum* has shown strain-targeting potential in tea fermentation, the stable application of *A. oryzae* and *Rhizopus* has been demonstrated in plant protein and grain matrix improvement, *Penicillium*’s cheese-like aroma has been shown to depend on fatty acid precursor matching, and basidiomycetes are becoming an important resource for the high value of natural aroma substances and by-products. These applications in traditional fermented foods have proved the practical significance of fungal aroma production.

## 7. Challenges and Prospects

Although significant progress has been made, the wide application of fungal aroma production still faces several challenges. The first is that the molecular mechanism of controlling aroma formation has not been clearly defined. Most of the current research remains at the level of identifying volatile compounds, while the network regulating key metabolic pathways still requires further exploration. Secondly, the diversity of aroma-producing fungi that have been systematically studied is limited. Many potentially valuable species have not been explored for aroma production, and there is a lack of genetic engineering tools, which limits the improvement of strains. Then, the transition from laboratory- to industrial-scale production faces major challenges, especially for the SSF process. Although SSF has the advantages of high substrate utilization efficiency and strong ecological adaptability of fungi, it is difficult to control heat and mass transfer, water content, and online monitoring and may require higher water consumption and more complex downstream separation. A comprehensive evaluation of water consumption across the entire life cycle should be performed when comparing SSF and SMF, including substrate pretreatment, fermentation, and downstream separation. Finally, comprehensive toxicological evaluations on fungal aroma products are still lacking. Food-grade fungi do not mean that all processes, substrates, and metabolites are naturally safe. Attention must be paid to the potential toxins in plant by-products, mycelium ingredients, and fermented beverages due to bacterial contamination, residual substrates, and metabolic by-products. Increases in volatile compounds do not necessarily entail sensory improvements. Aroma release, taste, color, texture, nutrition, and consumer acceptance should be included in a unified evaluation system; food matrix effects on aroma retention/release and risks of undesired off-flavor formation should also be considered [124,125]. Similarly, the ability to produce aroma is not equivalent to food safety. For genera such as Aspergillus, Penicillium, Monascus, and less studied basidiomycetes, specific safety assessments must be performed on the strains, including the screening of mycotoxin biosynthetic gene clusters, secondary metabolite analysis, allergenicity screening, and toxicological testing [126,127]. Relevant regulatory frameworks include the European Food Safety Authority’s (EFSA) Qualified Presumption of Safety (QPS), the US Food and Drug Administration’s (US-FDA) Generally recognized as safe (GRAS), the EU’s Novel food authorization, and China’s list of microbial strains available for food and approval requirements for new food raw materials. The extent of safety assessment required for a candidate strain should be proportionate to the species, intended use, production process, history of safe use, and applicable regulatory framework.

For the above problems and challenges, more in-depth research should be established, should be further developed, and should be systematically conducted in the following four directions. First, we should focus on elucidating the molecular mechanisms controlling aroma formation. Mechanism analysis using multi-omics techniques can be combined with genomics, transcriptomics, metabolomics, and proteomics; then, pure-culture, synthetic community, multi-omics, isotope tracing, aroma recombination, and omission experiments should be carried out to enhance the strength of causal evidence. At the same time, the regulatory network of key aroma-active compounds and the identification of key enzymes should be further explored.

Secondly, a library of food-grade fungal strains should be established, with classifications by enzyme activity, target aroma, safety, and substrate suitability. In particular, the systematic screening of excellent strains of basidiomycetes, *Rhizopus*, and *Aspergillus* should be strengthened. It is also necessary to continuously explore new aroma-producing strains and explore agricultural by-products as fermentation substrates, in line with the principle of circular economy. At the same time, genetic tools for strain improvement should also be developed, such as for the genetic engineering of high-yield strains using CRISPR and other gene-editing tools or for the heterologous expression of key enzyme genes.

Then, the process engineering research of SSF and SMF should be promoted. In this regard, AI can be used to assist the optimization of fermentation process parameters and flavor prediction, and electronic nose applications and near-infrared spectroscopy can be used for real-time monitoring to realize the control of intelligent fermentation processes. In addition, it is necessary to promote the industrial application of multi-omics and machine learning; establish datasets across batches, production areas, and process conditions; and incorporate temperature, humidity, pH, acidity, oxygen, matrix composition, microbial community status, and volatile spectrum into a unified modeling framework. For SSF systems such as liquor, vinegar, tea, and soybean products, spatial sampling and micro-environmental monitoring are key to improving accuracy.

Finally, a standardized safety evaluation of fungal aroma products should be established, including the assessment of mycotoxin risk, allergenicity, and potential toxicological effects. A safety evaluation process from genome to product should be established for candidate strains, including the screening of toxin synthesis gene clusters, screening of resistance genes and pathogenic factors, monitoring of secondary metabolites during fermentation, and toxicology and allergy risk assessment of final products. For objects with high application potential such as *Monascus*, *Aspergillus*, and *Rhizopus*, it is necessary to clarify the strain preservation number, whole-genome information, and fermentation conditions.

In conclusion, only by solving the problems of yield, stability, safety, regulation, and real-food sensory evaluation can fungal fermentation (filamentous fungi and basidiomycetes) move from laboratory aroma discovery to sustainable applications in the food industry. With the continuous progress of multi-omics technology, synthetic biology, and process engineering, we can expect to meet the growing demand of consumers for natural, sustainable, and clean-label flavor ingredients.

## Figures and Tables

**Table 1:** Main fungi and their aroma functions.

Fungal Groups	Representative Species	Typical Base Substance	Key Enzyme	Main Aroma Compounds	References
*Aspergillus*	*A. oryzae*	Cereals, broad beans	Amylase, glucoamylase, protease	Aldehydes, esters, organic acids	[33]
	*A. niger*	Pumpkin peel	Phenolic acid converting enzyme	Vanillin	[38,39]
	*A. cristatus*	White tea, black tea	Amylase, protease, laccase	Esters, linalool, benzaldehyde, β-ionone, 1-octen-3-ol	[40]
*Rhizopus*	*R. arrhizus*	Rice water	Amylase, protease, esterase	Esters, alcohols, organic acids	[46]
	*R. oligosporus*	Buckwheat	Glucoamylase, protease	Esters, pyrazines, organic acids, alcohols	[25,43]
*Penicillium*	*P. roqueforti* *P. camemberti*	Soy milk	Lipase	2-heptanone, 2-nonanone	[48]
Basidiomycete	*Pleurotus sapidus*	Citrus lateral flow	Dehydrogenase	P-anisaldehyde	[50]
	*Mycetinis scorodonius*	Green tea	Oxidoreductase, hydrolase	Isovaleraldehyde, linalool, geraniol, 2-phenylethanol	[49]
	*Wolfiporia cocos*	Black currant marc	Oxidoreductase, hydrolase	Benzaldehyde, vanillin, benzyl alcohol	[51]
	*Trametes versicolor* *Pycnoporus sanguineus*	Pineapple waste	Laccase, peroxidase	Aroma precursor conversion	[60]
*Monascus*/*Neurospora*	*Neurospora crassa*	Bean dregs	Protease, amylase, esterase	Organic acids, alcohols, esters	[35]
	*Monascu* spp.	Daqu	Protease, amylase, lipase	Alcohols, aldehydes, acids, esters, pyrazines, furans, phenols, and sulfur compounds	[58,59,61]

**Table 2:** Typical volatile compounds and their fermentation forms.

Typical Aroma Compounds	Flavor	Precursor/Substrate	Key Fungi	Fermentation Form	References
1-Octen-3-ol	Mushrooms, soil	Linoleic acid	*P. ostreatus*	SSF/SMF	[81]
1-Octen-3-one	Mushroom, metallic note	Linoleic acid	*P. ostreatus*	SMF	[81]
3-Octanone	Mushrooms, herbal	Linoleic acid	*P. ostreatus*, *P. camemberti*	SSF/SMF	[14,81]
2-Heptanone	Blue cheese, fatty	Mid-chain fatty acid	*P. roqueforti*	SSF	[48]
2-Nonanone	Blue cheese, fatty-floral	Mid-chain fatty acid	*P. roqueforti*	SSF	[48]
p-Anisaldehyde	Fennel, woody-herbal	L-tyrosine	*Pleurotus sapidus*	SMF	[50]
Vanillin	Herbal, sweet	Ferulic acid, pumpkin phenolic acid	*A. niger*	SSF	[36,38]
2-Phenylethanol	Rose, floral	L-phenylalanine	*A. aurulenta*	SMF	[52]
2-phenylethyl acetate	Honey-sweet, floral	L-phenylalanine	*A. aurulenta*	SMF	[52]
3-Methylbutan-1-ol	Malt, cocoa, nutty	L-leucine	*P. ostreatus*, *A. oryzae*	SSF/SMF	[81]
Linalool	Floral, citrus	Terpene precursors, tea matrix	*A. cristatus*	SSF	[39,40]

Note: Flavor descriptors were retrieved from Flavornet, The Good Scents Company, and the originally cited literature. Compounds listed in this table have been confirmed as aroma-active through GC–O, aroma recombination, or omission experiments in at least one of the cited references.

## Data Availability

No new data were created or analyzed in this study. Data sharing is not applicable to this article.
